# Role of Inflammatory Markers as a Risk Factor for Community-Acquired Pneumonia Management

**DOI:** 10.3390/medicina61061078

**Published:** 2025-06-11

**Authors:** Ruta Nutautiene, Irmantas Aleksa, Ieva Janulaityte, Erika Skrodeniene, Kristina Bieksiene, Diana Zaliaduonyte, Darius Batulevicius, Astra Vitkauskiene

**Affiliations:** 1 Department of Laboratory Medicine, Faculty of Medicine, Academy of Medicine, Lithuanian University of Health Sciences, A. Mickeviciaus st. 9, LT-44307 Kaunas, Lithuania; ruta.nutautiene@lsmu.lt (R.N.); ieva.janulaityte@lsmu.lt (I.J.); erika.skrodeniene@lsmu.lt (E.S.); 2 Centro Polyclinic, Naujamiestis Primary Personal Health Care Centre, Pylimo st. 3, LT-01117 Vilnius, Lithuania; irmantas.aleksa@centropol.lt; 3 Department of Pulmonology, Faculty of Medicine, Academy of Medicine, Lithuanian University of Health Sciences, A. Mickeviciaus st. 9, LT-44307 Kaunas, Lithuania; kristina.bieksiene@lsmu.lt; 4 Lithuanian University of Health Sciences Kaunas Hospital, Josvainiu st. 2, LT-47144 Kaunas, Lithuania; diana.zaliaduonyte@lsmu.lt; 5 Department of Cardiology, Faculty of Medicine, Academy of Medicine, Lithuanian University of Health Sciences, A. Mickeviciaus st. 9, LT-44037 Kaunas, Lithuania; 6 Institute of Anatomy, Academy of Medicine, Lithuanian University of Health Sciences, A. Mickeviciaus st. 9, LT-44307 Kaunas, Lithuania; darius.batulevicius@lsmu.lt

**Keywords:** community-acquired pneumonia, inflammatory cytokines

## Abstract

*Background and Objectives*: Community-acquired pneumonia (CAP) remains a major health burden worldwide, with high morbidity and mortality, particularly among older adults and those with comorbidities. This study aimed to evaluate the etiological factors of CAP and to investigate systemic inflammatory markers (IL-6, IL-8, IFN-*γ*, and G-CSF) in blood samples collected from CAP patients to identify which markers could be targets for potential etiological, clinical, and therapeutic interventions. *Materials and Methods*: A prospective study was conducted in 41 patients with confirmed CAP hospitalised during the winter season of 2024–2025. Clinical, demographic, and laboratory data were collected at admission and seven days later. Serum IL-6, IL-8, IFN-*γ*, and G-CSF concentrations were measured using a multiplex assay. *Results*: Aetiology was identified in 87.8% of cases, with typical bacterial pathogens being more prevalent among older, smoking patients, while atypical pathogens were more common among younger, non-smoking patients. Hospitalisation and increased inflammatory markers were associated with older age. After seven days of treatment, significant decreases in IL-6, IFN-*γ*, and G-CSF concentrations were observed. IFN-*γ* levels were significantly higher in patients with atypical aetiology. Higher concentrations of IL-8 and G-CSF were associated with hospitalisation. IL-6 levels were positively correlated with age, C-reactive protein (CRP), and pneumonia severity index (PSI) scores. *Conclusions*: Systemic inflammatory markers, especially IL-6, IL-8, IFN-*γ*, and G-CSF, may be valuable tools in managing generalised pneumonia. They can help to differentiate etiologically, assess disease severity, and make treatment decisions.

## 1. Introduction

Community-acquired pneumonia (CAP) is a significant problem not only in patients with risk factors, but for healthy people as well, and remains a major health concern because it is associated with a deadly condition. CAP’s high incidence and mortality are usually related to older age and comorbidities [1]. According to the World Health Organisation, pneumonia remains a leading cause of mortality worldwide, accounting for approximately 2.5 million deaths in 2019 across all age groups, with a particularly high burden in children under five and older adults [2]. Clinical studies show different CAP incidence data in many countries, but the real clinical incidence of CAP is challenging to determine because of differences in reporting and case selection from epidemiological studies. The mortality rate in hospitalised adults with CAP ranges from 5–15% and can be up to 30% in severe cases, especially in ICU settings or among the elderly [3]. In three South American cities, a high incidence rate of community-acquired pneumonia (CAP) in adults was identified, ranging from 1.76 to 7.03 per 1000 person-years, while, in Ireland, the hospitalisation rate for CAP was reported at 20.6 cases per 10,000 adults per year, and data from France showed an incidence of 4.7 per 1000 persons annually [4,5,6]. The presence of a lung infiltration on chest radiography, along with various symptoms such as cough, fever, dyspnoea, and chest pain, can be observed. Still, the diagnosis of CAP is sometimes difficult. CAP can be caused by respiratory viruses; atypical intracellular bacteria like *Legionella pneumophila* (*L. pneumophila*), *Chlamydophila pneumoniae* (*C. pneumoniae*), or *Mycoplasma pneumoniae* (*M. pneumoniae*); and typical bacterial pathogens like *Streptococcus pneumoniae* (*S. pneumoniae*), *Haemophilus influenzae* (*H. influenzae*), and *Staphylococcus aureus* (*S. aureus*). CAP aetiology is changing owing to pneumococcal and influenza vaccines. The role of viruses in CAP pathogenesis is not fully explained. Further studies are needed to improve outcomes in patients with CAP [1].

The analysis of systemic inflammatory response is important for understanding the pathogenesis of pneumonia, especially in patients with underlying disease.

First and foremost, we need to establish the derivations of immune response to different pathogens. Pneumococcus (*Streptococcus pneumoniae*) elicits an immune response primarily through the complement system. However, it has been observed that the immune response to pneumococcus can be modulated through the pneumococcus surface proteins pneumolysin and polyamine D transfer protein (PoDT). Pneumolysin derivatives (PdT and PoDT), when combined into a single subcutaneous vaccine, were successful in enhancing the body’s immune response to pneumococcus before exposure to pneumococcal infection [7,8]. The results showed that the combination of PdT and PoDT increased the amounts of specific antibodies in the body and prompted the secretion of inflammatory markers—IL-1*β*, IL-6, and TNF-*α* [9]. The study was conducted in mice, but this is likely to have a similar effect in humans, as identical immune reactions are induced [8].

Mycoplasma pneumoniae—a common causative agent of atypical CAP—are bacteria that do not have a wall, so they modulate the immune response differently than typical pathogens [10]. Their pathogenic molecular patterns (PMPs) are lipoproteins and glycolipids, which are recognised by dendritic cells and macrophage receptors; in particular, toll-like receptor (TLR)2, TLR3, TLR7, TLR8, and TLR9 may contribute to the recognition processes of mycoplasma PMPs and have a significant impact on the further expression of inflammation or the development of complications [11,12]. The immune response induced by mycoplasmas is best characterised by changes in IFN-*γ* concentrations, as this chemokine specifically activates the production of inflammatory cytokines (IL-6, IL-8, TNF-α, CXCL9), which increase the nonspecific immune response of neutrophils and monocytes via the alternative pathway [13,14]. A specific immune response is also possible through antigen-presenting cells. As a result, a large amount of M (IgM), and later G, class immunoglobulins (IgG) begin to be secreted. Specific immune responses occur using T lymphocytes (CD4+) when the body encounters the pathogen. The specific toxin of community-acquired respiratory distress syndrome (CARDS) has been better studied. The toxin acts through the NLRP3 inflammasome and further induces the expression of IL-1β and IL-18, which subsequently increases IL-6 and IL-8 and further increases IL-1β expression [15]. Indeed, the relationship between IFN-*γ*, CXCL9, and the CARDS toxin is not completely clear, although it has been found that reducing the amounts of CARDS secreted by mycoplasmas can undoubtedly modulate the immune response in patients with pneumonia [16,17].

Chlamydophila pneumoniae is the other common atypical pneumonia pathogen. The immune system responses to this pathogen are different because chlamydia do not have peptidoglycans in their cell walls and are obligate intracellular pathogens that infect many cells of the immune system, including monocytes and macrophages [18]. Chlamydia-induced immune responses are usually associated with humoral responses via T lymphocytes. However, the primary expression of inflammation is manifested through NK cells and IFN-*γ* secretion [19]. It has been established that the immune response to chlamydia infections is modulated through TLR2, TLR3, and TLR4 receptors through recognition of their PMP—heat shock protein (HSP60). Chlamydial infections, as usual, induce a primary immune response through the expression of TNF-*α*, IL-1*β*, IL-6, and IL-8 [20,21]. CD8+ cells receive the signal through MHC I. Upon the activation of CD8 cells, CD8+ cells induce IFN-*γ* secretion and perforin production. Perforin-deficient organisms responded to chlamydia-induced inflammation as rapidly as normal organisms. Nevertheless, IFN-*γ* secretion is the most important factor in the management of chlamydial infection. However, the immune response can be suppressed through programmed death ligand (PD-L1). If PD-L1 expression is suppressed on dendritic or epithelial cells, or if PD-L1 is eliminated, then IFN-*γ* secretion and chlamydia clearance are accelerated [19,22].

It is known that procalcitonin (PCT) levels rise in proportion to the severity of the bacterial infection, but the levels do not increase in viral infections. Therefore, low PCT levels preclude the need for antibiotics. PCT levels are associated with an increased rate of bacteraemia, septic shock, multi-organ failure, and mortality [23]. Calprotectin may represent a new marker of both bacterial aetiology and 5-year all-cause mortality, while plasma pentraxin 3 (PTX3) and presepsin are potential novel predictors of short-term outcome in CAP. The elevated calprotectin and white blood cell (WBC) levels in the convalescent phase may reflect unfavourable persistent inflammation associated with the increased risk of long-term mortality [24]. Pro-inflammatory and anti-inflammatory cytokines are important mediators in the host response to infection [25]. It is known that interleukin-6 (IL-6) is promptly and transiently produced in response to infections and tissue injuries and contributes to host defence through the stimulation of acute phase responses, haematopoiesis, and immune reactions. Rosolowski’s results suggest that elderly patients have a decrease in IL-6 concentration and an increase in thrombocytes, IL-6, and interleukin-8 (IL-8) concentrations associated with CAP severity [26]. Interferon-gamma (IFN-*γ*), granulocyte colony-stimulating factor (G-CSF), and IL-6 are multifunctional cytokines that regulate immune responses and cell proliferation, which frequently have functionally opposing roles, so the aim of our study was to determine the concentration of systemic inflammatory markers (Il-6, IL-8, IFN-*γ*, and G-CSF) in blood samples taken from patients with CAP that could serve as targets for potential aetiology, clinical, and therapeutic interventions.

## 2. Materials and Methods

### 2.1. Inclusion and Exclusion Criteria

The study included patients using these criteria:

- Age ≥ 18 years;
- Diagnosed with community-acquired pneumonia;
- Provided written informed consent.

Exclusion criteria were as follows:

- Age under 18;
- Inability to provide informed consent;
- Patients with autoimmune diseases;
- Patients with chronic lung diseases such as cystic fibrosis or COPD;
- Contraindications to venipuncture.

### 2.2. Patients and Study Design

A total of 41 patients with confirmed CAP during radiological examination (new infiltrate) and with at least two clinical symptoms (temperature > 38 °C, productive cough, crackles on auscultation, shortness of breath, chest pain), as long as they did not meet the exclusion criteria involved in this prospective study. Patients were treated or received consultation during the 2024–2025 winter season in the Kaunas Hospital of the Lithuanian University of Health Sciences. The exclusion criteria were for all study participants as follows: patients younger than 18 years, immunosuppressive and/or corticosteroid treatment, antibiotic treatment started more than 24 h before admission, and pregnancy. The study was approved by the Kaunas Regional Biomedical Research Ethics Committee on 28 March 2024, No. BE-2-1, and written consent from all subjects was received (Supplementary Materials). Smoking status was obtained using a standard questionnaire and ascertained by self-reported data. Non-smokers were defined as never smokers; otherwise, the subjects were classified as smokers (past and current smokers were included in the same group).

Clinical data and blood samples were collected on admission and day 7. The first patient visit was at the hospital in the case of hospitalised patients or in the outpatient setting in ambulatory cases. The questionnaire was used to collect demographic data and information on lifestyle habits (smoking cigarettes past or present), comorbidities (chronic obstructive pulmonary disease, diabetes mellitus, immunosuppression including cancer or medications, status, neurological disorders, chronic kidney or liver diseases). The severity of illness was assessed by the PSI score. A PSI score of 0 points was considered as risk class I, 1–69 points—risk class II, 71–90 points—risk class III. Patients were considered to have severe CAP if their PSI was 91–130 points, i.e., risk class IV; if higher, risk class V.

Blood for complete blood count (CBC), C reactive protein (CRP), PCT, microbiological sputum culture for typical bacteria before antibiotic treatment, and swab for respiratory viruses (Adenovirus, Rhinovirus/Enterovirus, Coronavirus 229E, Coronavirus 43, Coronavirus NL63, Coronavirus HKU1, SARS-CoV-2, Influenza A, Influenza B, Influenza A subtype H1N1/2009, Parainfluenza 1-4, Human metapneumovirus A/B, Respiratory syncytial virus A/B, Bocavirus) and atypical bacteria (*M. pneumoniae*, *C. pneumoniae*, *L. pneumophila*, *Bordetella pertussis*) were taken on the day of admission. Serum for atypical bacteria-specific M class immunoglobulin (IgM) testing was taken on day 7 after confirming the CAP diagnosis.

Sputum samples for typical bacteria detection were inoculated directly onto 5% sheep blood agar (Becton, Dickinson and Compan, Franklin Lakes, NJ, USA), chocolate agar (Becton, Dickinson and Compan, Franklin Lakes, NJ, USA), and Mac Conkey agar plates (Oxoid, UK). Sheep blood and chocolate agar plates were incubated at 35 °C in an atmosphere containing 5% CO_2_ and MacConkey agar plates—at 35 °C for 18–24 h. If culture was negative at the first observation, sheep blood and chocolate agar plates were re-examined after a second 24 h incubation [27]. A MALDI-TOF MS analyser (Bruker, Billerica, MA, USA) was used to identify pathogens.

For the detection of respiratory viruses and atypical bacteria DNA, a multiplex real-time PCR assay was used (FLASH Dx Respiratory Panel, Shenzhen, China), along with an Automated Nucleic Acid Detection System (FLASH Dx, Shenzhen, China).

### 2.3. Blood Sampling and Determination of Serum Concentration of Inflammatory Markers

Blood samples for inflammatory markers testing were drawn from each study patient when CAP was confirmed and after 7 days of treatment. Each blood sample was centrifuged, and serum was stored at −80 °C until testing. The serum concentrations of 4 cytokines—IL-6, IL-8, IFN-*γ*, and G-CSF—were quantified using a commercially available magnetic bead-based multiplex assay (ProcartaPlex, Invitrogen, Thermo Fisher Scientific, MA, USA) and a Luminex VR 100 Analyser (Luminex Corp., Austin, TX, USA), according to manufacturer instructions. Each sample was analysed in triplicate.

### 2.4. Statistical Analysis

Statistical data analysis was performed using IBM SPSS Statistics 30.0. The Kolmogorov–Smirnov test was employed to determine if quantitative data were distributed normally. The Mann–Whitney U and Kruskal–Wallis tests were used if data were not normally distributed. The Wilcoxon signed-rank test was used with related samples. Results were presented as the median and interquartile range (M (IQR)). The Chi-square, z-tests, and Fisher’s exact test (for small sample sizes) were used to determine relationships between qualitative data. Correlations were estimated using Spearman’s rank correlation test. Differences comparing the groups were considered statistically significant when the *p* value was less than 0.05.

## 3. Results

### 3.1. Characteristics of the Study Population

A total of 41 patients with CAP in 2024–2025, aged 22–93 years, were included in the analysis, with a mean age of 59.15 ± 21.63 years. In all, 13 (31.8%) study subjects reported that they were current or past smokers. Aetiology was established for 36 patients (87.8%); typical bacteria were found in 22 patients (*S. pneumoniae*-7, *H. influenzae*-9, *Enterobacterales*-5, *S. aureus*-1), and atypical bacteria or respiratory viruses in 14 (*M. pneumoniae*-10, *C. pneumoniae*-2, *Rhinovirus*-2). More elderly patients were found to have typical bacterial pathogens compared to atypical etiological agents, age median 71.5 (27) vs. 41 (41) years, *p* = 0.03. Typical bacterial aetiology has been established in 63.6% (*n* = 14) and atypical only in 21.4% (*n* = 3) in patients aged over 65 years (*p* = 0.04). Atypical pathogens were found more often in patients 65 years or younger, in contrast to patients older than 65 years: 78.6%, *n* = 11, and 36.4%, *n* = 8, respectively, *p* = 0.04. A significantly higher percentage of atypical pathogens was found in the never-smoking patients’ group compared to the smoking patients: 46.4%, *n* = 13, and 7.7%, *n* = 1, respectively, *p* = 0.040. Smoking patients more often have a typical bacterial pathogen compared to atypical or unknown aetiology (76.9%, *n* = 10, versus 7.7%, *n* = 1, and 15.4%, *n* = 2, respectively, *p* = 0.04). Additionally, 25 (61%) study subjects required hospitalisation due to their clinical condition and laboratory findings. Hospitalisation associated with patients’ age: age median 73 (38) vs. 49 (38), *p* = 0.043. There was no correlation between clinical signs, symptoms, laboratory parameters, and CAP aetiology, except WBC count: WBC count was higher in patients with CAP with unknown aetiology compared with typical bacterial agents, 11 (6.61) × 10^9^/L and 6.9 (3.16) × 10^9^/L, respectively, *p* = 0.026. Patients’ demographic, clinical, and laboratory data, depending on aetiology at the time of admission, are presented in Table 1.

### 3.2. Serum Inflammatory Marker Levels

To characterise the inflammatory status of study patients, we assessed the serum levels of 4 (IL-6, IL-8, IFN-*γ*, and G-CSF) markers on admission and day 7. The IL-6, IFN-*γ*, and G-CSF concentrations after 7 days of treatment significantly decreased compared to the concentration found on the 1st day, accordingly, 7241 (12,114) vs. 2195 (9362.8) pg/mL, *p* = 0.021; and 2445 (5877.9) vs. 0.83 (2444.3) pg/mL, *p* = 0.004; and 14,129.5 (41,837) vs. 7.0 (3890.8) pg/mL, *p* < 0.001. Data is presented in Table 2.

**Figure 1:**
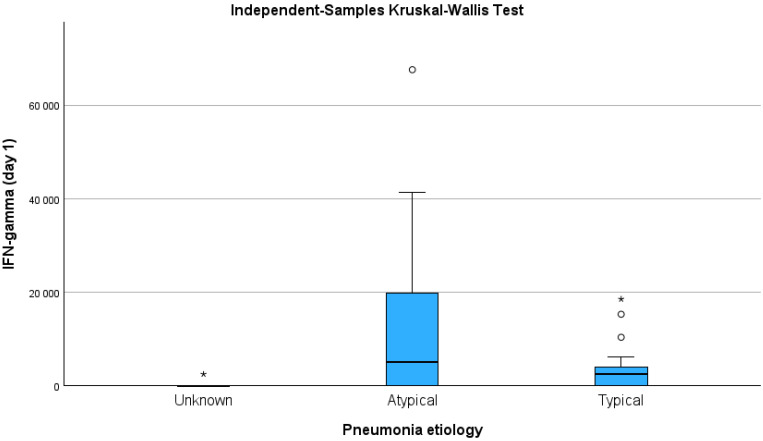
The IFN-*γ* concentration according to CAP aetiology; *p* < 0.05. * *p* < 0.05 compared to atypical pneumonia etiology.

The IFN-*γ* concentration was significantly higher in patients with atypical pneumonia compared to those with an unknown aetiology, accordingly, 4998.5 (20,913) vs. 0.7 (1222.6) pg/mL, *p* = 0.029 (Figure 1). Concentrations of other tested inflammatory markers did not differ depending on aetiology.

IL-8 and G-CSF concentrations were associated with the treatment setting: higher concentrations were found in patients treated in the hospital compared to ambulatory stay, accordingly, 34,763.5 (38,799.5) vs. 14,696.5 (24,524.5) pg/mL, *p* = 0.001; and 27,964 (36,832.5) vs. 7.0 (20,470) pg/mL, *p* = 0.013. IL-6 and IL-8 concentrations after 7 days of treatment were significantly higher in hospitalised patients in comparison with those treated as outpatients, accordingly, 3653.5 (10,066.8) vs. 727.6 (2744) pg/mL, *p* = 0.029; and 22,166.5 (32,024.25) vs. 12,876 (13,766.75) pg/mL, *p* = 0.017 (Table 3).

### 3.3. Integration of Clinical and Laboratory Data

Correlation between systemic inflammatory markers and clinical or laboratory data shows a positive correlation between IL-6 and age (*r* = 0.385; *p* = 0.015), IL-6 and CRP (*r* = 0.461; *p* = 0.003), IL-6 and PSI score (*r* = 0.346; *p* = 0.031), IL-8 and CRP (*r* = 0.348; *p* = 0.028), IFN-*γ* and CRP (*r* = 0.398; *p* = 0.011), G-CSF and CRP (*r* = 0.396; *p* = 0.011), and G-CSF and neutrophils (*r* = 0.328; *p* = 0.0039). Negative correlation was found between IFN-*γ* and WBC (*r* = −0.350; *p* = 0.027), and G-CSF and lymphocytes (*r* = −0.328; *p* = 0.042) (Table 4).

## 4. Discussion

Cytokines are critical mediators of both innate and acquired immune responses. In response to pathogens, cytokine functions include cell differentiation, chemotaxis, and the activation or regulation of pro- and anti-inflammatory processes [28,29,30]. We found that the IFN-*γ* concentration was significantly higher in patients with atypical pneumonia compared to those with pneumonia of unknown aetiology; however, IL-6 level did not differ significantly based on aetiology. The same results were found in Ding’s study [31]. IFN-*γ* levels were significantly elevated in atypical pneumonia, suggesting its potential as a differentiating biomarker, whereas IL-6 shows no significant variance across aetiologies. It only proposes the conclusion that IL-6 can be used in the assessment of immune response rather than in the differentiation of pathogens, thus highlighting IFN-*γ*’s critical role in lung immunity, which can be extrapolated to pneumonia contexts.

Previous studies have demonstrated distinct cytokine profiles and biomarker patterns, depending on the underlying cause of infection [32,33,34,35,36]. Different cytokine profiles and biomarkers were found, depending on the cause in the Menendez study: atypical bacteria (lower PCT and IL-6), *Enterobacteriaceae* (higher IL-8), *S. pneumoniae* (high PCT), and *Legionella pneumophila* (higher CRP and TNF-*α*). A prospective study of patients admitted with CAP showed higher levels of CRP, PCT, TNF-*α*, and IL-6 in those with bacteraemia compared to CAP of an unknown cause, but this was not considered secondary to bacteria [37]. IL-6 and IL-8 are essential mediators in influenza pneumonia [38]. Endeman reported that the systemic cytokine response is influenced by the causative microorganism and that corticosteroid treatment might reduce the cytokine levels in patients with CAP. Interestingly, genotype did not significantly influence the levels of cytokine production in CAP patients [39]. Cytokine and biomarker profiles vary depending on the pathogen, which can inform proficient diagnostic and therapeutic strategies.

Immune dysregulation during acute lung infection contributes to lung injury and a systemic inflammatory response, thus, cytokines play a major role in severe cases. In our study, IL-8 and G-CSF concentrations were associated with the treatment setting: higher concentrations were found in patients whose clinical condition required hospitalisation. Fernandez-Botran in his study evaluated circulating cytokines and results indicate that patients with severe CAP fail to mount a robust local pro-inflammatory response but exhibit instead a more substantial systemic inflammatory response, suggesting that a key driver of CAP severity may be the ability of the patient to generate an optimal local inflammatory response [40]. Other Fernandez-Botran results suggest the feasibility of using cytokine data at hospital admission to help identify those patients at higher risk. After further validation, such an approach may facilitate a better understanding of the relationship between local and systemic inflammatory responses and a more individualised therapeutic approach to modulate the inflammatory response in hospitalised patients with CAP [41]. Antunes’ study has demonstrated elevated circulating levels of TNF-*α*, IL-6, and IL-1ra on admission in most patients with CAP and a temporal pattern of systemic cytokine expression [25].

Systemic levels of IL-6, IL-10, and IFN-*γ* were significantly higher in severe CAP patients than in non-severe CAP patients and healthy individuals in Paats’s study [42]. Our study shows that IL-6 concentrations after 7 days of treatment were significantly higher in patients treated in the hospital compared to ambulatory patients. Serum IL-6 correlated best with both disease-specific and generic severity scores in Antunes’ study, and the authors concluded that IL-6 may therefore be a valid marker of inflammation in CAP [25]. Martin-Loeches’ study demonstrated that treatment failure correlated with IL-6 levels. There was a good correlation of IL-6 levels and CAP failure, and IL-6 and PCT with late CAP failure [43]. Raised systemic levels of IL-6 and IL-10 cytokines have been associated with poorer outcomes in community-acquired pneumonia in the Martinez study as well. Different independent factors were related to an excess of IL-6 and IL-10. Severe CAP might often be characterised by a diminished local but exaggerated systemic inflammatory response, underlining the need to evaluate both compartments for comprehensive assessment. Elevated IL-6 and IL-10 levels are linked to poor clinical outcomes and higher mortality, reinforcing their prognostic significance in CAP.

Confusion, hypotension, pleural effusion, and bacteraemia were associated with the inflammatory profile with the highest mortality rate, whereas anti-pneumococcal vaccination and previous antibiotic treatment appeared to be protective factors [44]. Tripon data demonstrated that IL-6 is a predictive marker for adverse outcomes alone. Studies have found a correlation between IL-6 and IL-10, IL-6 and IL-17A, and IL-10 and IL-17A. Serum levels of IL-1*β*, IL-6, TNF-*α*, and IL-10 have decreased significantly in dynamic cases, while IL-4 increases. IL-6 discriminates alone between adverse and favourable outcomes [45]. The marked increase of IL-6 serum levels during the acute phase makes it a potential biomarker of pneumococcal infection among children with CAP [46]. Dynamic changes and correlations among IL-6, IL-10, IL-17A, and other cytokines support their integrated role in immune regulation and outcome prediction.

In conclusion, our findings support the growing body of evidence that cytokine profiles are closely associated with the clinical course and severity of community-acquired pneumonia.

Elevated levels of IL-6, IL-8, IFN-*γ*, and G-CSF were found to correlate with disease severity, treatment setting, and clinical outcomes. These results highlight the potential utility of inflammatory cytokines as biomarkers for early risk stratification, prognosis, and therapeutic monitoring in CAP.

Our study had several limitations. First, the sample size was relatively small: only 41 patients. This makes it difficult to draw firm conclusions or apply the results to a wider population. The fact that it was conducted at a single centre also limits its generalisability. Clinical practices and common pathogens may vary between regions, so the findings may not be consistent with studies in other countries.

Another issue is the exclusion of patients with COPD or autoimmune diseases. These groups often have more severe forms of pneumonia, so their exclusion narrows the relevance of the results. The follow-up period was also short, just seven days. This may not be enough to understand the full picture, especially when assessing long-term outcomes or persistent inflammation.

There are also several methodological issues. Smoking status was self-reported, which may introduce bias. Although the study measured systemic cytokine levels, it did not assess local lung inflammation, which plays a key role in the disease. Without this local information, our understanding of the immune response remains incomplete.

Finally, the study did not examine viral load or functional aspects of immune activity. To gain a clearer picture of how the immune system responds to different types of CAP, future studies should be larger in scale, including more patients, more centres, and longer follow-up periods. This would also help to combine systemic and local data to provide a more complete picture.

The results of this study support the growing body of evidence that systemic inflammatory cytokines, particularly IL-6, IL-8, IFN-*γ*, and G-CSF, are closely associated with the clinical course, severity, and treatment setting of community-acquired pneumonia. The increased levels of these markers, especially in hospitalised patients, suggest their potential role in early risk stratification and disease monitoring. Notably, IL-6 showed consistent correlations with CRP, age, and PSI score, suggesting its potential as a reliable marker for assessing disease severity and prognosis. Meanwhile, IFN-*γ* was significantly higher in cases of atypical aetiology and may therefore have diagnostic value.

However, these observations also raise important questions for future studies. The study was limited to systemic markers, but local immune responses in the lungs likely play a crucial role in determining outcomes. Future studies should investigate the relationship between systemic and local cytokine responses to better understand the pathophysiology of community-acquired pneumonia. Bronchoalveolar fluid sampling or the use of non-invasive methods to assess local immune activity could help to elucidate this interaction.

Furthermore, although our study identified trends in cytokine levels over seven days, longer follow-up is needed to assess how these markers change throughout illness and recovery and how they may predict long-term outcomes or complications. Increasing the cohort size and including a more diverse patient population, particularly those with chronic lung disease or immunosuppression, would improve the generalisability and clinical relevance of the findings.

The role of coinfections and functional personal immune profiling also remains to be foreseen. Advanced techniques such as immune cell phenotyping or gene expression analysis would be useful to deepen the knowledge of individual immune response and help personalise treatment strategies.

In conclusion, while this study confirms the utility of systemic cytokine profiling in CAP, future studies should aim to validate these markers in larger, multicentre cohorts, link systemic and local immune responses, and explore their integration into standardised clinical algorithms for diagnosis, prognosis, and treatment optimisation.

The results of this study suggest that the measurement of systemic inflammatory cytokines, particularly IL-6, IL-8, IFN-*γ*, and G-CSF, could be a valuable aid in the clinical management of community-acquired pneumonia. These biomarkers have shown associations with disease severity, aetiology, and treatment setting, which may aid clinicians in early risk assessment and decision-making. For example, elevated IL-6 levels correlated with PSI scores and CRP, thus supporting its use as a marker of disease severity. IFN-*γ* has shown potential to differentiate atypical from typical pneumonia, providing diagnostic value in cases where microbiological results are equivocal or delayed. The ability to more accurately assess the inflammatory response may also aid in decisions regarding hospitalisation, the duration of antibiotic use, and a closer monitoring of the need. Incorporating cytokine profiling into routine clinical practice, once validated in larger studies, could improve personalised care, optimise resource allocation, and ultimately improve outcomes for patients with community-acquired pneumonia.

## 5. Conclusions

The inflammatory markers IL-6, IL-8, IFN-*γ*, and G-CSF may provide valuable insights for managing CAP. Elevated IL-8 and G-CSF concentrations were associated with the requirement for hospitalisation based on patients’ clinical conditions and laboratory findings. Moreover, IL-6 concentration after 7 days of treatment stays higher in hospitalised patients compared to those treated on an outpatient basis. These findings suggest that inflammatory cytokines could help in early risk stratification, disease progression monitoring, and tailoring therapeutic strategies in patients with CAP.

## Figures and Tables

**Table 1:** Patients’ demographic, laboratory, and clinical characteristics at the time of admission.

Characteristic	CAP of Unknown Aetiology, *n* = 5 *n* (%)	Typical Bacterial Pathogen, *n* = 22 *n* (%)	Atypical Pathogen, *n* = 14 *n* (%)	*p* Value
Age (M (IQR))	57 (38)	71.5 (27) *	41(41) *	0.030
>65 years	2 (40)	14 (63.6) *	3 (21.4) **	0.040
≤65 years	3 (60)	8 (36.4) *	11 (78.6) **	
Smokers, *n* (%)				
Current or past (*n* = 13)	2 (15.4) *^,^**	10 (76.9) *	1 (7.7) **	0.040
Never (*n* = 28)	3 (10.7) *^,^**	12 (42.9) *	12 (46.4) **	
Place of treatment, *n* (%)				
Ambulatory	2 (40)	9 (40.9)	5 (35.7)	1.000
Hospital	3 (60)	13 (59.1)	9 (64.3)	
Duration of treatment days Mean (SD)	5.7 (1.1)	8.3 (2.7)	8.6 (2.2)	0.193
PSI score, *n* (%)				
Risk class I	2 (40)	4 (18.2)	4 (28.6)	0.551
Risk class II−III	3 (60)	17 (77.3)	8 (57.1)	
Risk class IV−V	0 (0)	1 (4.5)	2 (14.3)	
Signs and symptoms, *n* (%)				
Fever	3 (75)	15 (68.2)	11 (84.6)	0.663
Cough	4 (100)	19 (86.4)	13 (100)	0.484
Sore throat	3 (75)	12 (54.5)	9 (69.2)	0.628
Dyspnoea	3 (75)	14 (63.6)	8 (61.5)	1.000
Headache	0 (0)	4 (18.2)	3 (23.1)	0.852
Sputum	4 (100)	19 (86.4)	10 (76.9)	0.678
Laboratory parameters				
WBC count (×10^9^/L)	11 (6.61) *	6.9 (3.16) *	7.82 (7.09)	0.026
Platelet (×10^9^/L)	223 (207.5)	271.5 (85.5)	256.5 (100.3)	0.451
Neutrophil count (×10^9^/L)	6.76 (6.39)	5.55 (4.06)	6.52 (5.28)	0.370
Lymphocyte count (×10^9^/L)	1.43 (0.89)	1.14 (1.11)	1.37 (0.93)	0.688
CRP (mg/L)	11 (84)	70.5 (136)	93.5 (144)	0.107

CAP—community-acquired pneumonia; CRP—C-reactive protein; WBC—white blood cells. * *p* < 0.05 compared to atypical pathogen; ** *p* < 0.05 compared to typical bacterial pathogen.

**Table 2:** The serum levels of inflammatory markers on admission and day 7.

Inflammatory Markers	Day 1 M (IQR) pg/mL	Day 7 M (IQR) pg/mL	*p* Value
IL-8	24,225.0 (28,861.0)	18,849 (22,285.0)	0.382
IL-6	7241.0 (12,114.0)	2195 (9362.8)	0.021
IFN-*γ*	2445.0 (5877.9)	0.83 (2444.3)	0.004
G-CSF	14,129.5 (41,837.0)	7.0 (3890.8)	<0.001

G-CSF—granulocyte colony-stimulating factor; IL—interleukin; IFN-*γ*—interferon *γ*; IQR—interquartile range; M—median. Data presented as median and interquartile range.

**Table 3:** The serum levels of inflammatory markers on admission and day 7 according to the treatment setting.

Inflammatory Markers		Inpatient Treatment	Outpatient Treatment	*p* Value
IL-8 M (IQR) pg/mL	Day 1	34,763.5 (38,799.5)	14,696.5 (24,524.5)	0.001
	Day 7	22,166.5 (32,024.3)	12,876.0 (13,766.8)	0.017
IL-6 M (IQR) pg/mL	Day 1	8658.0 (12,114.0)	2928.0 (10,757.8)	0.234
	Day 7	3653.5 (10,066.8)	727.6 (2744.1)	0.029
IFN-*γ* M (IQR) pg/mL	Day 1	2445.0 (10,747.2)	2445 (3868.3)	0.420
	Day 7	0.8 (4325.3)	0.83 (1410.3)	0.141
G-CSF M (IQR) pg/mL	Day 1	27,964.0 (36,832.5)	7.0 (20,470.0)	0.013
	Day 7	7.0 (7124.0)	7.0 (0.0)	0.652

IL-8—interleukin 8; IL-6—interleukin 6; IFN-*γ*—interferon gamma; G-CSF—granulocyte colony-stimulating factor; pg/mL—picograms per millilitre; IQR—interquartile range; M—median. Data presented as median and interquartile range.

**Table 4:** Correlation between systemic inflammatory markers and clinical or laboratory data.

	IL-8		IL-6		IFN-γ		G-CSF	
	*r*	*p*	*r*	*p*	*r*	*p*	*r*	*p*
Age	–0.008	0.960	0.385 *	**0.015**	–0.288	0.072	0.023	0.888
CRP	0.348 *	**0.028**	0.461 **	**0.003**	0.398 *	**0.011**	0.396 *	**0.011**
WBC	0.111	0.496	0.186	0.257	−0.350 *	**0.027**	0.175	0.280
PLT	−0.197	0.224	−0.065	0.692	0.033	0.842	−0.080	0.626
PSI score	0.188	0.244	0.346 *	**0.031**	0.114	0.483	0.167	0.303
IL-8	1.000	–	0.110	0.507	0.174	0.283	0.189	0.244
IL-6	0.110	0.507	1.000	–	−0.110	0.506	0.305	0.059
IFN-*γ* (1 day)	0.174	0.283	−0.110	0.506	1.000	–	0.259	0.107
G-CSF	0.189	0.244	0.305	0.059	0.259	0.107	1.000	–
Lymphocytes	−0.135	0.414	−0.112	0.503	−0.291	0.072	−0.328 *	**0.042**
Neutrophils	0.021	0.897	0.214	0.191	−0.185	0.253	0.328 *	**0.039**

* Correlation is significant at *p* < 0.05. ** Correlation is significant at *p* < 0.01. *r*—Spearman correlation coefficient; CRP—C reactive protein; WBC—white blood count; PLT—platelets count; PSI—Pneumonia Severity Index; IL-8—interleukin 8; IL-6—interleukin 6; IFN-*γ*—interferon gamma; G-CSF—granulocyte colony-stimulating factor.

## Data Availability

This article includes all the data presented in this study.

## Supplementary Materials

The following supporting information can be downloaded at: https://www.mdpi.com/article/10.3390/medicina61061078/s1, File S1: Approval to Conduct the Study; File S2: Informed Consent Form (in Lithuanian Language).

## Abbreviations

The following abbreviations are used in this manuscript.

CAP	Community-acquired pneumonia
CBC	Complete blood count
CRP	C reactive protein
G-CSF	Granulocyte colony-stimulating factor
IL	Interleukin
IFN	Interferon
PCT	Procalcitonin
PSI	Pneumonia severity index
