# Supplementary material for: Role of Inflammatory Markers as a Risk Factor for Community-Acquired Pneumonia Management
Source: Medicina (Kaunas). 2025 Jun 11;61(6):1078. doi: 10.3390/medicina61061078 (PMC12195302; doi:10.3390/medicina61061078)
Supplement: Supplementary file 1 [file medicina-61-01078-s001.zip › File S2.pdf]

PATVIRTINTA
Lietuvos bioetikos komiteto biomedicininių tyrimų ekspertų grupės 2016 m. lapkričio 15 d. sprendimu
PAKEISTA
Lietuvos bioetikos komiteto biomedicininių tyrimų ekspertų grupės 2020 m. birželio 16 d. sprendimu



---

# INFORMUOTO ASMENS SUTIKIMO FORMA

**Biomedicininio tyrimo pavadinimas:** Pneumonijos sukėlėjų patogeniškumo veiksnių ir paciento uždegimo žymenų prognostinė vertė ir sąsajos su ligos eiga bei išeitimi

**Protokolo Nr.:** Pneumonija-1

**Užsakovas:** Lietuvos sveikatos mokslų universitetas

**Adresas:** Adomo Mickevičiaus g. 9, 44307, Kaunas, Lietuva,

**Tel.:** +370 37 327201 **El. paštas:** rektoratas@lsmu.lt

**Užsakovo atstovas:** Prorektorė mokslui prof. habil. dr. Vaiva Lesauskaitė

**Atsakingas tyrėjas**[1]**:** Doc. dr. Kristina Biekšienė, LSMU ligoninės Kauno klinikų Pulmonologijos klinikos gydytoja pulmonologė

**Tyrimo centro pavadinimas:** Lietuvos sveikatos mokslų universiteto ligoninės Kauno klinikos

**Adresas:** Eivenių g. 2, 50161, Kaunas, Lietuva

**Tel.:** +370 37 326953 **El. paštas:** rastine@kaunoklinikos.lt

**Atsakingas tyrėjas**[2]**:** Rūta Nutautienė, Lietuvos sveikatos mokslų universiteto Kauno ligoninės gydytoja pulmonologė

**Tyrimo centro pavadinimas:** Lietuvos sveikatos mokslų universiteto Kauno ligoninė

**Adresas:** Hipodromo g. 13, 45130, Kaunas, Lietuva

**Tel.:** +370 37 306 000 **El. paštas:** info@kaunoligonine.lt

---

[1] *Atsakingas tyrėjas – tyrimo metu konkretų pacientą, pasirašantį Informuoto asmens sutikimo formą, prižiūrėsiantis tyrėjas. Nurodomi jo kontaktai.*

[2] *Atsakingas tyrėjas – tyrimo metu konkretų pacientą, pasirašantį Informuoto asmens sutikimo formą, prižiūrėsiantis tyrėjas. Nurodomi jo kontaktai.*

**1. Kokia šio dokumento paskirtis?**

Šioje formoje pateikiama Jums skirta informacija apie biomedicininį tyrimą „Pneumonijos sukėlėjų patogeniškumo veiksnių ir paciento uždegimo žymenų prognostinė vertė ir sąsajos su ligos eiga bei išeitimi“, aptariamos tyrimo atlikimo priežastys, mokslinio tyrimo procedūros, nauda, rizika, galimi nepatogumai ir kita svarbi informacija. Jei nuspręsite dalyvauti tyrime, prašysime Jūsų pasirašyti šią sutikimo formą, kuria sutinkate dalyvauti tyrime. Pasirašydami šį dokumentą, patvirtinate sutikimą dalyvauti (leisti dalyvauti) moksliniame tyrime. Neskubėkite ir atidžiai perskaitykite šį dokumentą, jei nesupratote kokio nors žodžio ar teiginio, visus iškilusius klausimus būtinai užduokite gydytojui ar kitiems tyrimo komandos nariams. Prieš priimdami sprendimą, galite pasitarti su šeimos nariais, draugais ar savo gydytoju.

**BIOMEDICININIO TYRIMO TIKSLAS**

**2. Kodėl atliekami biomedicininiai tyrimai?**

Biomedicininio tyrimo metu Jums bus atliekamas sveikatos patikrinimas ar medicininės procedūros, kurios iš esmės nesiskiria nuo įprastos (kasdienės) klinikinės praktikos. Įprastos (kasdienės) klinikinės praktikos tikslas yra Jus (t. y. konkretų asmenį, pacientą) išgydyti ir/ar pagerinti Jūsų sveikatos būklę, o biomedicininio (mokslinio) tyrimo tikslas – atlikti epidemiologinį medicinos įstaigų darbuotojų tyrimą. Kitaip tariant, pagrindinis šio tyrimo tikslas nėra tiesioginė nauda Jūsų sveikatai.

**3. Kodėl atliekamas šis tyrimas?**

Šio tyrimo tikslas: nustatyti uždegimo žymenų prognostinę vertę bei pneumonijos sukėlėjų patogeniškumo veiksnių, paciento rizikos veiksnių ir imuninio atsako žymenų raiškos sąsajas su ligos eiga ir išeitimis. Šis tyrimas padės kompleksiškai įvertinti pneumonijos (plaučių uždegimo) išsivystymo ir skirtingos klinikinės eigos priežastis.

**BIOMEDICININIO TYRIMO PLANAS**

**4. Kokie asmenys pasirenkami dalyvauti šiame tyrime?**

Kviečiame Jus dalyvauti tyrime, nes sergate pneumonija (plaučių uždegimu) ir atitinkate pagrindinius įtraukimo į tyrimą kriterijus. Pagrindiniai įtraukimo į šį tyrimą kriterijai yra šie:

- 18 m. amžiaus ir vyresni asmenys, kuriems bus nustatyta visuomenėje įgytos pneumonijos diagnozė arba dėl įvairių priežasčių stacionare gydomi pacientai, kuriems bus nustatyta hospitalinės pneumonijos diagnozė Lietuvos sveikatos mokslų universiteto ligoninės Kauno klinikose ar Lietuvos sveikatos mokslų universiteto Kauno ligoninėje.
- Asmenys, sutinkantys savanoriškai dalyvauti tyrime.
- Asmenys, perskaitę ir pasirašę informuoto sutikimo formą.

- neįtraukimo į biomedicininį tyrimą kriterijai:
- Asmenys, kuriems kontraindikuotina ar galima komplikuota venos punkcijos procedūra.
- Sergantys autoimuninėmis ligomis
- Sergantys lėtinėmis plaučių ligomis, tokiomis kaip cistinė fibrozė, lėtinė obstrukcinė plaučių liga ir kt.

Pagal anamnezę gydytojas pulmonologas vertins simptomų pradžios laiką, taigi ir sirgimo pneumonija laiką. Pagal Lietuvos Higienos institutą, hospitaline infekcija laikoma ta infekcija, kuria pacientas suserga po 48 val. nuo patekimo į gydymo įstaigą.

**5. Kas atlieka/užsako šį biomedicininį tyrimą?**

Šio biomedicininio tyrimo užsakovas yra Lietuvos sveikatos mokslų universitetas. Tai viena didžiausių medicinos mokymo ir mokslo įstaiga, kurios struktūriniuose padaliniuose – ligoninės Kauno klinikų ir Kauno ligoninės skyriuose yra vykdomas šis biomedicininis tyrimas ir priemonių, reikalingų tyrimui, išlaidas padengs Lietuvos sveikatos mokslų universitetas.

**6. Tikimybė patekti į skirtingas tiriamųjų grupes ir dalyvavimo šiose grupėse ypatybės**

Netaikoma.

**7. Kiek truks Jūsų dalyvavimas šiame tyrime?**

Jūs dalyvausite biomedicininiame tyrime tik šio vizito metu.

**8. Kokiose šalyse bus vykdomas šis tyrimas?**

Tyrimas bus atliekamas Lietuvoje.

**9. Kiek tiriamųjų dalyvaus numatyta šiame tyrime?**

Tikimasi, kad šiame tyrime dalyvaus 400 žmonių.

**BIOMEDICININIO TYRIMO METU TAIKOMI METODAI**

**10. Ką Jums reikės daryti?**

Prašysime Jūsų leisti šio tyrimo tikslais paimti veninio kraujo ėminį (2 x 5 ml) ir Jūsų serume ištirti imuninio atsako žymenis (IL-1, IL-6, IL-8, IL-10, IL-17, TNF, INF gama). Taip pat paprašysime užpildyti klausimyną apie Jūsų demografinius duomenis, gretutines ligas ir klinikinius simptomus. Paimti mėginiai ir surinkta informacija bus koduota, neleidžianti tiesiogiai nustatyti Jūsų tapatybės (detalesnė informacija apie duomenų saugojimą ir prieinamumą pateikiama šio dokumento 18 ir 19 punktuose). Jūsų medicininiais dokumentais nesinaudosime.

Sutikus dalyvauti tyrime, Jums nereikės papildomai atvykti ir atlikti jokių diagnostinių ar gydomųjų procedūrų.

**NUMATOMA BIOMEDICININIO TYRIMO NAUDA TIRIAMAJAM**

**11. Ar dalyvavimas biomedicininiame tyrime Jums bus naudingas? Kokios naudos galite tikėtis dalyvaudami šiame tyrime?** *(pateikti*) Dalyvaudami biomedicininiame tyrime pasitarnausite mokslo pažangai. Tiesioginės asmeninės naudos neturėsite.

**GALIMA BIOMEDICININIO TYRIMO RIZIKA IR NEPATOGUMAI**

**12. Su dalyvavimu šiame tyrime susijusi rizika ir nepatogumai**

Dalyvaudamas/-a šiame tyrime galite patirti nepatogumų, tokių kaip sugaištas laikas susipažįstant su biomedicininio tyrimo informacija, pasirašant *Informuoto asmens sutikimo dalyvauti biomedicininiame tyrime formą*, atsakant anketos klausimus ir paimant veninio kraujo ėminius. Numatoma sugaišto laiko trukmė – apie 45 minutes. Po 30 d. su Jumis bus susisiekta telefonu, pulmonologo konsultacijai, jei bus reikalinga pakartotinė kontaktinė konsultacija ir/ar atliekami papildomi diagnostiniai tyrimai - gydytojas pulmonologas registruos pakartotinei konsultacijai.

Be to, pirmojo vizito metu atliekama venos punkcijos procedūra, gali sukelti nemalonių pojūčių – galite jausti nedidelį skausmą ar nežymų laikiną diskomfortą.

Dėl nenumatytų aplinkybių konfidenciali informacija gali tapti prieinama tretiesiems asmenims, kuriems ją suteikti nebuvote davęs/-usi sutikimo.

**GALIMOS ŽALOS, PATIRTOS DĖL BIOMEDICININIO TYRIMO, ATLYGINIMO TVARKA**

**13. Informacija apie draudimą**

Tyrimo užsakovas ir pagrindinis tyrėjas apdrausti biomedicininio tyrimo užsakovo ir pagrindinio tyrėjo civilinės atsakomybės privalomuoju draudimu. Vadinasi, Jūs turite teisę į žalos sveikatai ir su tuo susijusios neturtinės žalos, patirtos dalyvaujant šiame tyrime, atlyginimą. Dalyvavimas šiame tyrime neturėtų sukelti nepageidaujamo poveikio Jūsų sveikatai.

**TIRIAMOJO TEISĖS**

**14. Kokias pasirinkimo galimybes turėsite, jeigu nesutiksite dalyvauti šiame tyrime arba atšauksite sutikimą jame dalyvauti?**

Tyrime dalyvaujate savanoriškai, todėl turite teisę atsisakyti, o pradėjęs galite iš jo pasitraukti iki kol nepaimti kraujo mėginiai. Jei sprendimas nedalyvauti tyrime Jūsų sveikatai keltų pavojų, gydytojas tyrėjas paaiškins, kaip tokiu atveju geriausia elgtis. Jūsų sprendimas atsisakyti dalyvauti ar nutraukti dalyvavimą tyrime nedarys jokios įtakos teikiamai įprastinei sveikatos priežiūrai.

**15. Ar galėsite nutraukti dalyvavimą tyrime?**

Be rašytinio Jūsų sutikimo Jūs negalite būti įtrauktas į tyrimą. Nepaisant rašytinio sutikimo dalyvauti tyrime, Jūs turite teisę nuspręsti pasitraukti iš tyrimo. Tai galėsite padaryti be apribojimų.

Jums nusprendus pasitraukti iš tyrimo pagrindinis tyrėjas paprašys parašyti laisvos formos atsisakymo prašymą arba užpildyti atsisakymo formą.

Norėtume atkreipti dėmesį, kad šio tyrimo rezultatai, t. y. tyrimo dokumentuose iki Jūsų sutikimo dalyvauti biomedicininiame tyrime atšaukimo įrašyti duomenys nebus sunaikinti, nes jie reikalingi tyrimo išlaidoms pagrįsti.

Jūs turite teisę nesutikti, kad biomedicininio tyrimo tikslu toliau būtų naudojama Jūsų sveikatos informacija, gauta šio biomedicininio tyrimo metu.

**16. Jūsų dalyvavimo tyrime nutraukimo aplinkybės ir kriterijai**

Jūsų dalyvavimas tyrime gali būti nutrauktas jei yra nepaimti mėginiai. Kitų aplinkybių ir kriterijų nėra.
Tėvai/globėjai ar kiti teisėtai įgalioti asmenys turi teisę atšaukti sutikimą tiriamajam dalyvauti tyrime, esant pripažintam neveiksnumui, apribotam veiksnumui ar dėl sveikatos būklės negalint vertinti savo interesų

**17. Ar dalyvaudami šiame tyrime patirsite kokių nors išlaidų?**

Dalyvaudami šiame biomedicininiame tyrime, tiesioginių išlaidų nepatirsite.
Už dalyvavimą biomedicininiuose tyrimuose atlygis nėra mokamas. Už sugaištą laiką atlygis taip pat nebus mokamas.

**SVEIKATOS INFORMACIJOS KONFIDENCIALUMO GARANTIJOS**

**18. Ar Jūsų asmens duomenys bus konfidencialūs?** ***(privalo būti visa informacija!)***

Biomedicininį tyrimą atliekant gauta sveikatos informacija, leidžianti nustatyti asmens tapatybę, yra konfidenciali ir gali būti teikiama tik pacientų teises ir asmens duomenų apsaugą reglamentuojančių įstatymų nustatyta tvarka.
Duomenų valdytojas yra Lietuvos sveikatos mokslų universitetas, įmonės kodas: 302536989, adresas A. Mickevičiaus g. 9, LT 44307 Kaunas. Tel.: +370 37 327201, Faksas.: +370 37 220733, El. paštas: rektoratas@lsmuni.lt
Siekiant apsaugoti duomenų konfidencialumą, Jums bus suteiktas specialus kodas, kuris bus nurodomas visuose dokumentuose, išskyrus sutikimo formą (šiame dokumente bus nurodyti Jūsų asmeniniai duomenys). Sąrašą, kuriame Jūsų vardas ir pavardė susiejami su kodu, saugos pagrindinis tyrėjas seife, į kurį prieigą turi tik jis ir įgaliotas tyrėjas.
Kompiuteriai, kuriuose saugomi elektroniniai tyrimo dokumentai ir duomenys, apsaugoti slaptažodžiu. Prisijungimo kodus žino tik tyrėjai. Visos patalpos turi patekimo į jas kontrolės sistemą.
Jei sutiksite dalyvauti šiame tyrime, tyrėjai naudos tyrimui atlikti reikalingus Jūsų asmeninius duomenis. Duomenys bus renkami remiantis Jūsų pateikta informacija.
Biomedicininį tyrimą atliekant gauta sveikatos informacija nelaikoma konfidencialia ir gali būti paskelbta be tiriamojo asmens sutikimo, jeigu paskelbus tokią sveikatos informaciją nebus galima tiesiogiai ar netiesiogiai nustatyti tiriamojo asmens tapatybės.

**19. Kas ir kokiu tikslu galės susipažinti su Jūsų asmens duomenimis?** ***(privalo būti visa informacija!)***

Pasirašydami šią formą sutinkate, kad tyrimo centro tyrėjai, tyrimus kontroliuojančios institucijos (tokios kaip Valstybinė vaistų kontrolės tarnyba, biomedicininių tyrimų etikos komitetai) ir įgalioti tyrimo užsakovo (Lietuvos sveikatos mokslo universiteto) tyrimą prižiūrintys asmenys galės susipažinti su visa šio tyrimo tikslais apie Jus surinkta informacija. Kitiems asmenims ar įstaigoms bus teikiami tik užkoduoti duomenys, neleidžiantys tiesiogiai nustatyti Jūsų tapatybės. („Užkoduoti“ reiškia, kad dokumentuose bus nurodomas ne Jūsų vardas ir pavardė, o specialus numeris, kurį susieti su Jūsų asmeniu galės tik tyrėjas).
Surinktus duomenis tyrėjai naudos tik šio klinikinio tyrimo tikslais. Užsakovas užkoduotus sveikatos duomenis gali naudoti atlikdamas tyrimą, pateikdamas prašymus dėl mokslinio tyrimo rezultatų įteisinimo,

ar norėdamas kurti diagnostikos ir/ar medicinines priemones. Užkoduoti Jūsų mėginiai bus apdorojami, saugojami iki tyrimo atlikimo ir tiriami LSMU Laboratorinės medicinos klinikoje . Jūsų tyrėjas turi šios laboratorijos kontaktinę informaciją – jei norėtumėte susipažinti su šia informacija kreipkitės į savo tyrėją. Jūs turite teisę susipažinti su surinktais Jūsų asmens duomenimis ir turite teisę reikalauti ištaisyti neteisingus, neišsamius, netikslius Jūsų asmens duomenis. Jums pasitraukus iš tyrimo tyrėjai nesinaudos Jūsų duomenimis, bet negalės sunaikinti iki tol surinktų duomenų

**20. Kiek laiko bus saugomi tyrimo metu surinkti duomenys ir kas už tai bus atsakingas?**

Visa informacija bus užrašoma specialiai klinikiniam tyrimui sudaromuose elektroniniuose ir popieriniuose dokumentuose ir tyrimo centre saugoma 10 metų pasibaigus tyrimui. Tiek laiko saugoti duomenis įpareigoja teisės aktai / užsakovo nustatyta tvarka / siekiant užtikrinti duomenų kokybę ir kontrolę. Vėliau Jūsų asmens duomenys bus sunaikinti tyrimo centro nustatyta tvarka. Už dokumentų saugojimą tyrimo centre bus atsakingas pagrindinis tyrėjas doc. Kristina Biekšienė ir Lietuvos sveikatos mokslų universiteto ligoninė Kauno klinikos (tyrimo centras 1) kartu su pagrindiniu tyrėju arba pagrindinis tyrėjas gyd. Rūta Nutautienė ir LSMU ligoninė (tyrimo centras 2).

**21. Kas įvertino šį biomedicininį tyrimą? / Į ką kreiptis, jeigu iškiltų klausimų?**

Jūs turite teisę bet kuriuo metu užduoti su tyrimu susijusius klausimus – nedvejodami kreipkitės į gydytoją-tyrėją ar kitą tyrimo komandos narį. Gydytojai- tyrėjai doc. dr. Kristinai Biekšienei galite paskambinti tel.: +370 37 326953; gydytojai-tyrėjai Rūtai Nutautienei galite paskambinti +370 670 97754.

Lietuvos sveikatos mokslų universiteto Kauno ligoninės Duomenų apsaugos pareigūnas:
Viktorija Bučinskaitė, Josvainių g. 2, Kaunas duomenuapsauga@kaunoligonine.lt Tel. 8 602 35 949
Lietuvos sveikatos mokslų universiteto ligoninės Kauno klinikų Duomenų apsaugos pareigūnas – Tomas Kuzmarskas, el. p. tomas.kuzmarskas@kaunoklinikos.lt, tel. +37037326268

Dėl savo, kaip tyrimo dalyvio teisių, galite kreiptis į Lietuvos bioetikos komitetą: adresas Vilniaus g. 16, LT-01402, Vilnius. Tel.: (8 5) 212 45 65, faks.: (8 5) 260 86 40. El. paštas: lbek@bioetika.sam.lt

Jei kyla klausimų dėl tyrimo teisėtumo ar dėl savo, kaip tiriamojo teisių, galite kreiptis į leidimus išdavusias institucijas (Lietuvos bioetikos komitetą arba atitinkamą regioninį biomedicininių tyrimų etikos komitetą), informaciją apie Lietuvoje vykstančius tyrimus galima rasti šių institucijų interneto svetainėse: http://bioetika.sam.lt

Leidimą Lietuvos sveikatos mokslų universitetui atlikti asmens duomenų tvarkymo veiksmus **išdavė Valstybinė duomenų apsaugos inspekcija,** A. Juozapavičiaus g. 6, LT-09310 Vilnius, tel. (8 5) 212 75 35, el. paštas: ada@ada.lt . Jūs turite teisę pateikti skundą dėl asmens duomenų tvarkymo Valstybinei duomenų apsaugos inspekcijai. Skundą galite pateikti paštu (adresu: L. Sapiegos g. 17, 10312 Vilnius) arba naudodamiesi Valstybinės duomenų apsaugos inspekcijos elektroninių paslaugų sistema: /go.php/lit/Prisijungti/37L. Valstybinės duomenų apsaugos inspekcijos kontaktinis telefono numeris (8-5) 212 75 32, el. paštas: ada@ada.lt .

**22. Kita svarbi informacija, kuri gali turėti įtakos Jūsų apsisprendimui sutikti ar atsisakyti dalyvauti biomedicininiame/ klinikiniame vaistinio preparato tyrime.** Nėra

## SUTIKIMAS DALYVAUTI BIOMEDICININIAME TYRIME

**Pneumonijos sukėlėjų patogeniškumo veiksnių ir paciento uždegimo žymenų prognostinė vertė ir sąsajos su ligos eiga bei išeitimis**

Aš perskaičiau šią Informuoto asmens sutikimo formą ir supratau man pateiktą informaciją.

Man buvo suteikta galimybė užduoti klausimus ir gavau mane tenkinančius atsakymus.

Aš supratau, kad galiu atsisakyti dalyvauti tyrime, nenurodydamas/-a priežasčių ir motyvų.

Supratau, kad norėdamas/-a atšaukti sutikimą dalyvauti biomedicininiame tyrime, raštu turiu apie tai informuoti pagrindinį tyrėją.

Patvirtinu, kad turėjau užtektinai laiko apsvarstyti man suteiktą informaciją apie biomedicininį tyrimą.

Supratau, kad dalyvavimas šiame tyrime yra savanoriškas.

Patvirtinu, kad sutikimą dalyvauti šiame biomedicininiame tyrime duodu laisva valia.

Leidžiu naudoti asmens duomenis ta apimtimi ir būdu, kaip nurodyta Informuoto asmens sutikimo formoje.

Patvirtinu, kad gavau Informuoto asmens sutikimo formos egzempliorių, pasirašytą tyrėjo / kito jo įgalioto biomedicininį tyrimą atliekančio asmens.

Asmuo (kitas sutikimą turintis teisę duoti asmuo)

| vardas | pavardė | atstovavimo pagrindas | parašas | pasirašymo data | pasirašymo laikas |
|---|---|---|---|---|---|

Patvirtinu, kad suteikiau informaciją apie biomedicininį tyrimą aukščiau nurodytam asmeniui.

Patvirtinu, kad asmeniui (ar kitam sutikimą duoti turinčiam teisę asmeniui) buvo skirta pakankamai laiko apsispręsti dalyvauti biomedicininiame tyrime, atsižvelgiant į biomedicininio tyrimo pobūdį, taip pat įvertinus kitas aplinkybes, galinčias daryti įtaką priimamam sprendimui.

Aš skatinau asmenį (ar kitą sutikimą turintį teisę duoti asmenį) užduoti klausimus ir į juos atsakiau.

Tyrėjas (kitas įgaliotas biomedicininį tyrimą atliekantis asmuo)

| vardas | pavardė | pareigos tyrime | parašas | pasirašymo data | pasirašymo laikas |
|---|---|---|---|---|---|
