# Supplementary material for: Role of Inflammatory Markers as a Risk Factor for Community-Acquired Pneumonia Management
Source: Medicina (Kaunas). 2025 Jun 11;61(6):1078. doi: 10.3390/medicina61061078 (PMC12195302; doi:10.3390/medicina61061078)
Supplement: Supplementary file 1 [file medicina-61-01078-s001.zip › File S1.pdf]

Pasirašyta kvalifikuotu elektroniniu parašu
GINTAUTAS GUMBREVIČIUS
Data: 2024-03-28 16:59:23 GMT+2

KAUNO REGIONINIS BIOMEDICININIŲ TYRIMŲ ETIKOS KOMITETAS

Lietuvos sveikatos mokslų universitetas, A. Mickevičiaus g. 9, LT 44307 Kaunas, tel. (+370) 37 32 68 89; el.paštas: kaunorbtek@lsmuni.lt

LEIDIMAS ATLIKTI BIOMEDICININĮ TYRIMĄ

2024-03-28 Nr. BE-2-1

**Biomedicininio tyrimo pavadinimas: „Pneumonijos sukėlėjų patogeniškumo veiksnių ir paciento uždegimo žymenų prognostinė vertė ir sąsajos su ligos eiga bei išeitimi“**	
Protokolo Nr.:	**Pneumonija-1**
Data:	**2024-01-29**
Versija:	**1.3**
Asmens informavimo forma	**Versija: 1.3; data: 2024-01-29**
Pagrindinis tyrėjas:<br>Biomedicininio tyrimo vieta:<br>Įstaigos pavadinimas:<br>Adresas:	**Doc. dr. Kristina Biekšienė**<br>Lietuvos sveikatos mokslų universiteto ligoninė Kauno klinikos<br>Pulmonologijos klinika<br>Eivenių g. 2, LT-50161, Kaunas<br>**Gyd. Rūta Nutautienė**<br>Lietuvos sveikatos mokslų universiteto Kauno ligoninė Vidaus ligų skyrius Pulmonologijos poskyris<br>Hipodromo g. 13, LT-45130, Kaunas

Išvada:
Kauno regioninio biomedicininių tyrimų etikos komiteto posėdžio, įvykusio **2024 m. kovo 4 d.** (protokolo Nr. 2024-BE10-0003) sprendimu pritarta biomedicininio tyrimo vykdymui.

Mokslinio eksperimento vykdytojai įsipareigoja: (1) nedelsiant informuoti Kauno regioninį biomedicininių tyrimų etikos komitetą apie visus nenumatytus atvejus, susijusius su studijos vykdymu, (2) iki sausio 15 dienos – pateikti metinį studijos vykdymo apibendrinimą bei, (3) per mėnesį po studijos užbaigimo, pateikti galutinį pranešimą apie eksperimentą, (4) užregistruoti biomedicininį tyrimą pasirinktoje tyrimų registracijos platformoje prieš įtraukiant pirmąjį tiriamąjį.

Kauno regioninio biomedicininių tyrimų etikos komiteto nariai			
Nr.	Vardas, Pavardė	Veiklos sritis	Dalyvavo posėdyje
1.	Doc. dr. Gintautas Gumbrevičius	Klinikinė farmakologija	Taip
2.	Prof. dr. Kęstutis Petrikonis	Neurologija	Ne
3.	Dr. Saulius Raugelė	Chirurgija	Ne
4.	Prof. dr. Lina Jankauskaitė	Pediatrija	Ne
5.	Prof. dr. Džilda Veličkienė	Endokrinologija	Taip
6.	Prof. dr. Eimantas Peičius	Visuomenės sveikata	Taip
7.	Aušra Degutytė	Visuomenės sveikata	Taip
8.	Doc. dr. Žydrūnė Luneckaitė	Visuomenės sveikata	Taip
9.	Viktorija Bučinskaitė	Teisė	Ne

Kauno regioninis biomedicininių tyrimų etikos komitetas dirba vadovaudamasis etikos principais nustatytais biomedicininių tyrimų Etikos įstatyme, Helsinkio deklaracijoje, vaistų tyrinėjimo Geros klinikinės praktikos taisyklėmis.

Kauno RBTEK pirmininkas — LIETUVOS SVEIKATOS MOKSLŲ UNIVERSITETAS * KAUNO REGIONINIS BIOMEDICININIŲ TYRIMŲ ETIKOS KOMITETAS — Doc. dr. Gintautas Gumbrevičius
